# Rapid, quantitative and ultra-sensitive detection of cancer biomarker by a SERRS-based lateral flow immunoassay using bovine serum albumin coated Au nanorods†

**DOI:** 10.1039/c9ra09471g

**Published:** 2020-01-02

**Authors:** Luchun Lu, Jiangliu Yu, Xiaoxian Liu, Xingsheng Yang, Zihui Zhou, Qing Jin, Rui Xiao, Chongwen Wang

**Affiliations:** College of Life Sciences, Anhui Agricultural University Hefei 230036 PR China jinqing169@126.com ruixiao203@sina.com wangchongwen1987@126.com; Beijing Institute of Radiation Medicine Beijing 100850 PR China; Anhui Provincial Key Laboratory of Veterinary Pathobiology and Disease Control, Anhui Agricultural University Hefei 230036 PR China

## Abstract

Early diagnosis of cancer biomarkers is the key to guiding treatments and improving the survival rate of patients. Herein, we report a novel surface-enhanced resonance Raman scattering (SERRS)-based lateral flow immunoassay (LFIA) for quantitative and ultra-sensitive analysis of alpha-fetoprotein (AFP). Gold nanorods (AuNRs) were fabricated to be in resonance with 785 nm laser excitation, that is, the excitation level that can maximize SERRS activity. The AuNRs were modified with 5,5′-dithiobis(2-nitrobenzoic acid), bovine serum albumin (BSA), and AFP detection antibody successively as the SERRS nanotags for the LFIA system. Modification of the BSA layer guaranteed good stability and biocompatibility of the SERRS nanotags in complex samples. The SERRS-LFIA strip for AFP detection showed a low detection limit of 9.2 pg mL^−1^ and a broad detection range from 10 pg mL^−1^ to 500 ng mL^−1^. By comparison, the detection limit of the proposed assay is about 100 and 10 times lower than those of the Au nanoparticle-based SERS-strip and conventional enzyme-linked immunosorbent assay, respectively. Moreover, the potential clinical applications of the assay were evaluated by detecting 10 actual serum samples. Results showed 100% accuracy based on the clinical tests.

## Abstract 2

A rapid, sensitive, and stable SERRS-LFIA strip was developed for AFP detection using BSA-coated AuNRs as SERRS nanotags.

## Graphical abstract

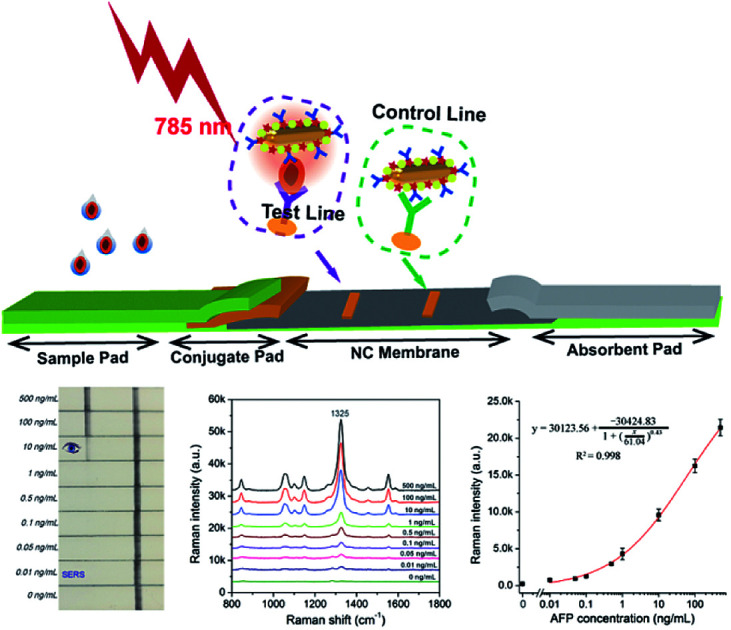

## 1. Introduction

Cancer is one of the most life-threatening diseases worldwide, with over 18.1 million new cases and approximately 9.6 million deaths reported in 2018.^1^ The symptoms of cancers do not usually appear until at the terminal stage, which results in high death rates.^2^ Therefore, the early diagnosis of cancer can guide active therapy and improve the survival rate of patients. Detection of biomarkers has become one of the most promising methods for the early diagnosis of cancer.^3^ Changes in cancer biomarker concentration in human biological specimens, such as serum, blood, urine, or saliva, are key indicators of tumor processes and treatment responses.^4,5^ The methods currently used to detect cancer biomarkers mainly include chemiluminescent immunoassay, enzyme-linked immunosorbent assay (ELISA), immuno-blotting, electrochemical immunoassay, and time-resolved fluorescence immunoassay.^6–10^ Although these methods are well elaborated and widely used in clinical laboratories, they still exhibit shortcomings, such as tedious operation process, long testing time, and the use of expensive equipment or unstable reagents, in real-time detection of biomarkers. These drawbacks have hindered their wide point-of-care testing (POCT) applications, particularly in low-resource settings, such as clinics, infirmaries, and homes. Thus, sensitive and convenient POCT methods must be developed for cancer biomarker diagnosis.

Lateral flow immunoassay (LFIA) has been considered one of the most promising POCT tools in bioanalysis fields because of its distinct advantages of simple operation, rapid analysis, low cost, and user-friendly format.^11–14^ However, the conventional LFIA strip, which uses colloidal gold as label and relies on colorimetric signal readout, possesses inherent limits, including low sensitivity and limited qualitative or semi-quantitative detection capability.^15,16^ Surface-enhanced Raman scattering (SERS) is an effective detection tool with high sensitivity and can address the limitations of the LFIA strip.^17–19^ By using SERS nanotags, SERS-based immunoassays have been successfully applied in the quantitative determination of various cancer biomarkers, including alpha-fetoprotein (AFP), prostate-specific antigen (PSA), carcinoembryonic antigen (CEA), cancer antigen (CA) 15-3 and CA 27-29, and exosomes.^20–26^ A typical SERS nanotag comprises Au/Ag nanoparticle (NP)-based enhanced substrate, loaded Raman reporter molecules, and surface-modified antibodies, thus ensuring the sensitivity, quantitative capability, and specificity of SERS-based immunoassays. Recently, several studies have shown that the combination of SERS nanotags and LFIA strips can realize the sensitive detection of specific protein toxins, cardiac biomarkers, inflammatory markers, and DNA markers.^27–29^ Most of these studies used Au, Ag, or Au@Ag nanospheres to build SERS nanotags. However, the maximum localized surface plasmon resonance (LSPR) of these SERS nanotags is between 400–600 nm, which is not optimal in the terms of SERS performance because of the mismatch between LSPR and the commonly used 785 nm excitation wavelength. The 785 nm laser is the most frequently used excitation wavelength for measuring biological samples; it can effectively reduce the damage to biological specimens and the autofluorescence background.^30–32^

Herein, we report a new surface-enhanced resonance Raman scattering (SERRS)-LFIA strip based on Au nanorods (NRs)-based SERRS nanotags for rapid, sensitive, and quantitative detection of cancer biomarkers in serum. Given that the LSPR of AuNR nanotags at 785 nm matches the provided excitation wavelength, the SERS activity of the nanotags further improved the resonance enhancement effect, resulting in higher detection precision and sensitivity.^33^ Moreover, considering that common Au/Ag-based SERS nanotags are easily affected by environmental factors (*e.g.*, salt ion strength and impurities), a layer of bovine serum albumin (BSA) was introduced onto the surface of the SERRS nanotags to ensure their high colloidal stability in biological samples. For demonstration, AFP, which is the most commonly used tumor biomarker for the diagnosis of hepatocellular carcinoma (HCC) and other malignancies, was selected as the model cancer biomarker.^34,35^ A pair of AFP monoclonal antibodies was separately conjugated on the AuNR nanotags and the test line of LFIA strip. In the presence of AFP antigens in the serum, sandwich immunocomplexes would form on the T line through antibody–antigen reactions. Hence, the SERRS signal of AuNR nanotags trapped on the test line can be easily recorded for the quantitative analysis of AFP. Under optimum conditions, the SERRS-LFIA strip can perform rapid detection of AFP with a low detection limit of 9.2 pg mL^−1^ and show excellent stability in fetal calf and human serum. Moreover, the proposed method exhibits higher sensitivity and wider detection range than the commercial ELISA method. To our knowledge, this study is the first to introduce AuNR nanotags to construct high-performance SERRS-LFIA strips. We believe that the proposed method shows considerable potential for cancer biomarker detection in the POCT settings.

## 2. Experimental

### 2.1 Chemicals and materials

Gold(iii) chloride trihydrate (HAuCl_4_), trisodium citrate, BSA, sodium hydroxide (NaOH), silver nitrate (AgNO_3_), and hydrochloric acid (HCl) were supplied by Sinopharm Chemical Reagent Co., Ltd (China). 5,5′-Dithiobis(2-nitrobenzoic acid) (DTNB), *N*-(3-dimethylaminopropyl)-*N*′-ethylcarbodiimide hydrochloride (EDC), sodium borohydride (NaBH_4_), hexadecyltrimethylammonium bromide (CTAB), l-ascorbic acid (AA), and Tween 20 were obtained from Sigma-Aldrich (USA). Glycine was purchased from Amresco (USA). Glutaraldehyde (50 wt% solution in H_2_O) was supplied by J&K Scientific Co., Ltd (China). Mouse anti-AFP antibodies (10-3140 and 10-3141) and AFP protein were obtained from Fitzgerald (USA). Goat anti-mouse IgG was purchased from Sangon Biotech Co., Ltd (China). Nitrocellulose (NC) membrane (Hi-Flow Plus HF135) was supplied by Millipore Corporation (USA). The sample loading pad, conjugate pad, absorbent pad, and polyvinyl chloride bottom plate were purchased from Jieyi Biotechnology Co., Ltd (China). The commercial human AFP ELISA kit (Catalog #KIT12177) was acquired from Sino Biological, Inc. (China).

### 2.2 Ethical statement

Human serum samples of HCC patients and healthy person were collected from the Department of Laboratory Medicine, Affiliated Hospital of Xuzhou Medical University, and stored at −80 °C until use. All experiments were conducted in accordance with the Declaration of Helsinki, and they were approved by the Ethics Committee of the Beijing Institute of Radiation Medicine and Affiliated Hospital of Xuzhou Medical University (approval ID: XYFY2018-KL110). Informed consents were obtained from human participants of this study.

### 2.3 Instruments

Transmission electron microscopy (TEM) images were taken on a H-7650 microscope (Hitachi, Japan) at an accelerating voltage of 80 kV. High-resolution TEM (HRTEM) images of AuNRs were taken on a Tecnai G2 F20 microscope (Philips, Holland) operated at 200 kV. Zeta potentials of various nanoparticles were examined by using a Nano-ZS90 ZetaSizer (Malvern, UK). UV-vis spectra were measured with a Shimadzu 2600 spectrometer. Raman spectrum of the tested strips were measured by a portable Raman system (B&W Tek, i-Raman Plus BWS465-785H spectrometer) with 785 nm laser excitation. All samples were excited with a power of 10 mW and a total acquisition time of 10 s for each SERS spectra.

### 2.4 Preparation of plasmonic AuNRs

AuNRs were synthesized according to our previously proposed seed-mediated growth method with several modifications.^36^ Briefly, the Au seed solution was prepared by dissolving 183 mg CTAB in 5 mL deionized water followed by adding 42 μL HAuCl_4_ (1%). Then, 300 μL freshly prepared NaBH_4_ solution (0.01 M) was added to the above solution under vigorous stirring (30 °C, 600 rpm) for 2 min. During the process, the color of the seed solution immediately changed to brownish yellow. After the magnetic stirring was halted, the seed solution was further aged for 1 h at 30 °C. To synthesize plasmonic AuNRs, 0.8 mL HAuCl_4_ (1%), 0.35 mL AgNO_3_ (10 mM), 0.4 mL HCl (2 M), and 0.32 mL AA (0.1 M) were added to 40 mL 0.1 M CTAB solution. The solution was then shaken gently until it became colorless. Afterward, 140 μL as-prepared seed solution was added to the growth solution, and the mixture was placed in 30 °C water bath overnight. The obtained AuNRs were further purified by washing them twice with deionized water to remove excess reagents and re-suspended in 10 mL deionized water for future use.

### 2.5 Preparation of AuNR-based plasmonic SERRS tags

**Scheme 1:**
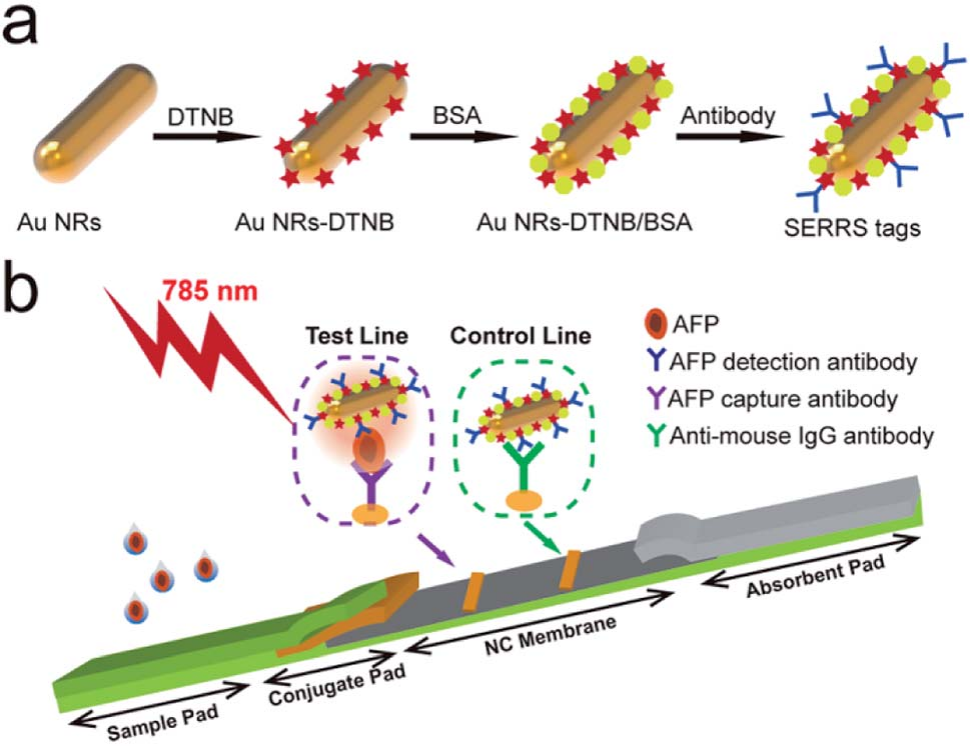
(a) Preparation process of BSA-coated SERRS tags. (b) Schematic of quantitative detection of AFP based on SERRS-LFIA strip.

The plasmonic SERRS tags were fabricated *via* a two-step coupling strategy. The first step was performed to prepare DTNB-modified AuNRs (AuNR-DTNB), and the second step conjugated the detection antibodies onto the surface of AuNR-DTNB (Scheme 1a). Briefly, 3 μL DTNB ethanol solution (10 mM) was added to 1 mL AuNRs and then ultrasonicated at room temperature for 1 h. The formed AuNR-DTNB solution was centrifuged at 7500 rpm for 6 min to remove the excess DTNB and then re-suspended in 1 mL deionized water. The conjugation of antibody and AuNR-DTNB was based on previously reported methods with slight modification.^37^ First, 100 μL BSA (1.0%) mixed with 2 μL glutaraldehyde (25%) was added to the pre-prepared AuNR-DTNB solution and shaken for 4 h. The supernatant was removed by centrifugation, and 1 mL of 10 mM glycine was added. Then, the sample was shaken for 30 min to remove excess aldehyde groups. The pellet was re-suspended in borate buffered saline (BBS) solution (10 mM, pH 8.0) after washing twice. Subsequently, the carboxyl groups at the end of BSA molecules were activated by addition of 100 μL of 10 mM EDC. After incubation for 20 min, excess activating agents were separated from the solution by centrifugation (7500 rpm, 6 min) and re-dispersed in BBS buffer (10 mM, pH 8.0). A total of 15 μg AFP detection antibody was added with activated AuNR-DTNB@BSA, and the mixture was shaken for 3 h. Finally, the unreacted carboxyl group was blocked with 100 μL of 10% BSA for 1 h. The precipitate was separated by centrifugation twice and then re-suspended in 100 μL buffer containing 10% sucrose, 6% d-trehalose, 2% BSA, 0.6% polyvinylpyrrolidone, and 1% Tween 20. The suspension was used as stock AuNR nanotags solution for further use.

### 2.6 Preparation of SERRS-LFIA strips

The SERRS-LFIA strip consisted of an NC membrane, a sample loading pad, a conjugate pad with plasmonic SERRS tags, and an absorbent pad (Scheme 1b). Goat anti-mouse (0.8 mg mL^−1^) and AFP-capture antibodies (0.8 mg mL^−1^) were sprayed on the NC membrane by a spraying platform (Biodot xyz5050) to form the control and test lines, respectively. Both antibodies were diluted with a coating buffer (10 mM phosphate-buffered saline (PBS); pH 7.4) and dispensed onto the NC membrane at a rate of 1 μL cm^−1^. The antibody-coated NC membrane was dried in a 37 °C incubator for 3 h. Finally, the SERRS-LFIA strip was assembled and cut into 3.0 mm wide strips and stored in a dry-sealed container.

### 2.7 Detection of AFP based on SERRS-LFIA strips

The AFP antigen was serially diluted from 500 ng mL^−1^ to 0.01 ng mL^−1^ in a running buffer as a positive sample, and PBS containing 1% Tween 20 and 20% fetal bovine serum (FBS) was used as a blank control to determine the sensitivity of the prepared SERRS-LFIA strips. A total of 70 μL sample containing different concentrations of antigen was dropped onto the sample pad of the prepared strips. The liquid migrated along the NC membrane to the absorbent pad by capillary action. After the test strips were dried, the SERS signals were recorded using a portable Raman spectrometer with a laser power of 10 mW and an integration time of 10 s.

ELISA was used as a traditional detection method to compare the sensitivity of the SERRS-LFIA strips. When the solution containing different concentrations of AFP antigen was added, the AFP in the solution was bound to the capture antibody onto the well. After incubation and washing three times, the horseradish peroxidase-conjugated detection antibody was added to bind to the AFP antigen, thereby forming the sandwich immune complex. After removing the unbound antibody, the substrate solution was added, and the reaction was finally terminated by the addition of a stop solution. The color of the solution gradually darkened from yellow as the concentration of AFP antigen increased, and the intensity was measured at 450 nm.

### 2.8 Detection of biological samples

Given that normal human blood also contains human AFP, we first used unprocessed fetal calf serum instead of human serum to detect AFP and to verify the sensitivity of the SERRS-LFIA strip and its capability for application to biological samples. AFP was diluted from 500 ng mL^−1^ to 0.01 ng mL^−1^ using FBS and added to the SERRS-LFIA strip. The Raman signal intensity was measured by a portable Raman spectrometer. For further analysis, 10 clinical serum samples of confirmed AFP concentrations were collected from Xuzhou Medical University Affiliated Hospital and tested using the SERRS-LFIA strip.

## 3. Results and discussion

### 3.1 Operating principle of the SERRS-LFIA strip

To achieve the goal of ultra-sensitive and quantitative detection of serum biomarkers, we proposed a novel SERRS-LFIA strip using BSA-coated AuNR nanotags in place of the commonly used AuNP-based nanotags. The fabrication of AuNR/BSA nanotags consists of four steps (Scheme 1a): (i) synthesis of a plasmonic AuNR as enhancing substrate, (ii) adsorption of a layer of DTNB molecules to produce strong and specific Raman signal, (iii) modification of a layer of BSA to obtain better stability in complex samples, and (iv) conjugation of monoclonal antibodies to identify the target biomarker. Here, we adopted AuNRs as substrate because of their strong Raman scattering enhancement owing to the localization of stable hot spots near the nanorod tips and their plasmon peak, which can be easily adjusted to match with the 785 nm excitation laser to generate the greatest plasmonic coupling effect.

Scheme 1b illustrates the general principle of AuNR-nanotag-based-LFIA strip, which is based on antibody–antigen–antibody sandwich-type immunoassay, for the quantitative and ultra-sensitive analysis of AFP. As the sample solution containing the AFP antigen was loaded onto the sample loading pad, the solution migrated toward the absorbent pad through capillary force. Afterward, the AFP antibody-conjugated AuNR nanotags on the conjugate pad recognized specifically the AFP antigen and were finally captured by the anti-AFP antibody on the test zone, thereby forming sandwich immunocomplexes through antibody–antigen reactions. Excess AuNR nanotags continually migrated and were then immobilized by the goat anti-mouse IgG antibodies on the control line. Consequently, two visible bands appeared when the AFP antigen was present, and one band of the control line was observed when the target was absent. Finally, the SERRS signal of the AuNR nanotags on the test line was measured for highly sensitive and quantitative detection of the target AFP using a 785 nm laser.

### 3.2 Characterization of AuNR-based SERRS nanotags

**Fig. 1:**
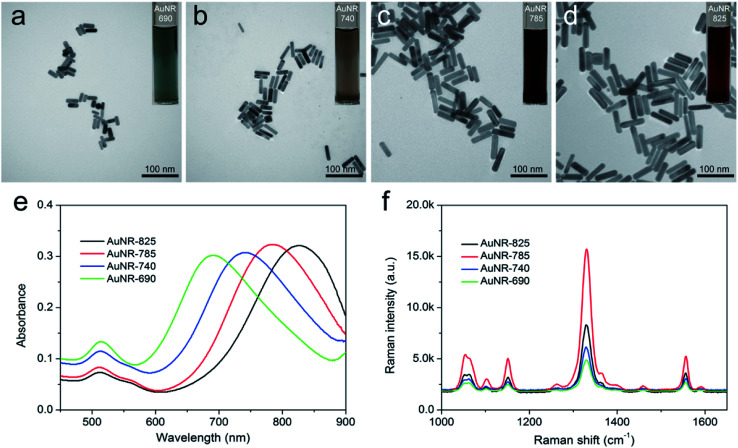
TEM images of AuNRs at different synthesis conditions: (a) 0.25, (b) 0.3, (c) 0.35, and (d) 0.4 mL of 10 mM AgNO_3_. The insets are their corresponding optical pictures. (e) UV-vis spectra of the obtained AuNRs with different aspect ratios. (f) SERS intensity of AuNR-DTNB with four different aspect ratios under the same conditions.

AuNRs are one of the most efficient SERRS substrates because their continuous LSPR can be easily adjusted to match the wildly used near-infrared incidence laser (785 nm), thus not only considerably improving the SERRS activity but also reducing background autofluorescence and sample damage for bioanalysis applications.^38,39^ In this study, 75 nm rod-shaped AuNRs were fabricated *via* a modified seed-mediated approach according to our previous work.^36^ As shown in Fig. 1a–d, AuNRs with different sizes could be well controlled by varying the concentration of AgNO_3_. All these synthesized AuNRs showed good shape and size uniformity. The UV-vis spectra of the AuNRs with different aspect ratios were measured, as shown in Fig. 1e. Notably, the LSPR band of the obtained AuNRs gradually red-shifted to the near-infrared region with the increasing AgNO_3_ concentration in the reaction system. The LSPR wavelengths of AuNRs with four different sizes, denoted as AuNR-690, AuNR-740, AuNR-785, and AuNR-825, were 690, 740, 785, and 825 nm, respectively. To compare their SERS activity directly, the synthesized AuNRs were modified with the same concentration of DTNB molecules under sonication for 2 h. As revealed in Fig. 1f, all the signature peaks of DTNB at 846, 1059, 1151, 1328, and 1555 cm^−1^ were observed and consistent with those reported in literature.^40^ Evidently, the SERS intensity of the AuNR-785, whose plasmon peak was in resonance with the 785 nm laser excitation wavelength, was about 2.1 times than that of AuNR-825 and more than 3 times than that of AuNR-690 and AuNR-740. The superior SERS activity of AuNR-785 can be attributed to the best SERRS response from the plasmonic coupling effect.^41,42^ Furthermore, the commonly used AuNP (50 nm) were synthesized as SERS nanotag, and its SERS activity was compared with that of AuNR-785. As shown in Fig. S1,† the SERS intensity of AuNR-785 is about 4.2 times than that of 50 nm Au NPs. In addition, their SERS enhancement factors (EF) for the main peak of DTNB (1328 cm^−1^) were roughly calculated by an adjusted method, as described in detail in ESI S1.† The results showed that the EF of AuNR-785 was estimated to be 1.45 × 10^6^.

**Fig. 2:**
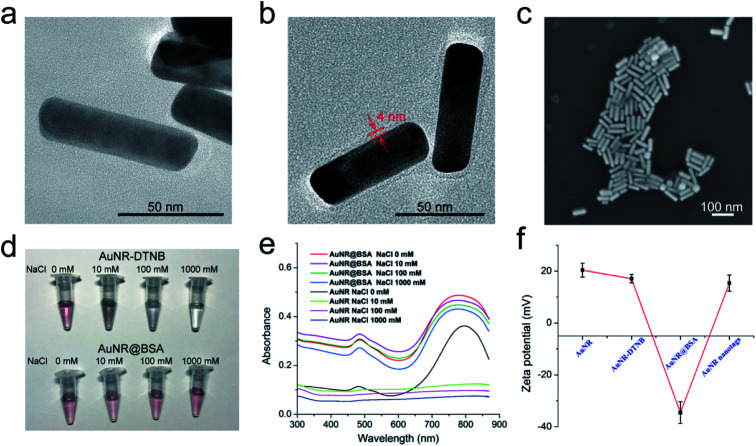
HRTEM images of (a) AuNR-DTNB and (b) AuNR@BSA. (c) SEM image of AuNR@BSA on Si chip. (d) Color changes and (e) UV-vis spectra of AuNR-DTNB and AuNR@BSA under varying salt concentrations (0–1000 mM NaCl). (f) Zeta potential of AuNR nanotags from each step.

Numerous studies indicated that antibody conjugation to CTAB-coated AuNRs is difficult because the CTAB layer would inhibit the coupling process, and the AuNRs would easily aggregate in the presence of high-salt buffer.^43^ To improve the stability of the AuNRs in the complex samples, we used BSA to modify the surface of the AuNR-DTNB and then conjugated it with the AFP detection antibody. A layer of BSA was introduced to coat the AuNRs by the crosslinking reaction between BSA and glutaraldehyde in accordance with A. Samanta's method.^44^ The successful coating of BSA was investigated by HRTEM. By comparing the HRTEM images of AuNR-DTNB and AuNR@BSA (Fig. 2a and b), the BSA layer, with a thickness of approximately 4 nm, was found to be distributed around the AuNR-DTNB. After the BSA layer was coated, the UV-vis spectra peaks were slightly blue-shifted, and the peak width became wider (Fig. S2a†). This phenomenon can be explained by the particle size distribution turned wider with the coating of the BSA layer. Fig. S2b† displayed the EDS elemental mapping result of AuNR@BSA. Dense S element (green) was located around the Au element (red) surface, which clearly demonstrated the core–shell nanostructure of AuNR@BSA. Additionally, the SEM image showed the good dispersity of the prepared AuNR@BSA (Fig. 2c). The zeta potential of AuNR-DTNB decreased remarkably from +17.1 mV to −34.5 mV after coating with BSA, indicating the formation of AuNR@BSA (Fig. 2f). Bare AuNR-DTNB and AuNR@BSA were mixed with different concentrations of NaCl solution to evaluate the stabilization effect of BSA coating. As shown in Fig. 2d, the color of the AuNR-DTNB suspension turned colorless when varying concentrations of NaCl solution (10–1000 mM) were added, whereas that of AuNR@BSA remained stable in extremely high concentration of salt solution (1000 mM). The UV-vis spectra of AuNR@BSA showed no considerable change at 1000 mM NaCl aqueous solution, also indicating the dispersion of AuNR@BSA (Fig. 2e). By contrast, the absorbance of AuNR-DTNB dispersion at 785 nm immediately disappeared when NaCl solution was added, demonstrating the serious aggregation of AuNRs. Fig. S3† shows the TEM observations of the AuNR-DTNB aggregation and AuNR@BSA in the NaCl solution. These results confirm the good stabilization capability of BSA coating, which is the key to guaranteeing the SERRS performance and stability of AuNR nanotags for complex sample detection. Moreover, the BSA layer can provide sufficient carboxyl groups on the surface of AuNRs for anti-AFP antibody bioconjugation. The zeta potential of the nanotags and the protein absorption of the reaction solution were measured to confirm the antibody conjugation on the AuNR@BSA surface. The zeta potential of AuNR@BSA notably increased with antibody conjugation from −34.5 mV to +15.4 mV (Fig. 2f). The original data of zeta potentials are provided in Fig. S4.† Moreover, UV absorption at 280 nm of the supernatants of nanotags markedly decreased after the antibody modification, also demonstrating that the AFP antibody was successfully modified on the AuNR nanotags (Fig. S5†). The effect of the amount of AFP antibody modified on the AuNR nanotags was also investigated. Herein, the AuNR nanotags were prepared by using excessive amount of antibody. The maximum load capacity of antibody on the AuNR-785 was investigated by incubating different amount of AFP antibody. The obtained AuNR nanotags were used in the SERRS based LFIA detection. As shown in Fig. S6,† the SERS signal of 15 μg AFP antibody modified AuNR nanotags was strong enough and stable. This result indicated that the surface of the AuNR nanotags has been saturated with antibody.

### 3.3 Optimization of SERRS-LFIA assay

The excellent stability and SERRS activity enable the AuNR nanotags as the high-performance nanotags for LFIA strip detection. To optimize the performance of SERRS-LFIA strip, the influence of running buffer, NC membrane, antibody concentration on test line, and amount of SERRS nanotags on the conjugate pad have been systematically studied. The running buffer of SERRS-LFIA system is first optimized, because it directly affects the flow rate of SERRS nanotags and the immune binding efficiency of the test line. PBS buffer (10 mM, pH 7.4) containing different concentration of Tween 20, and FBS were tested, because Tween 20 can ensure smooth delivery of AuNR nanotags along the strip, and FBS can reduce the nonspecific adsorption on the NC membrane.^45,46^ With the increase of Tween 20 content in PBS solution, the fluidity of the nanotags on the SERRS-LFIA strip are continuously improved. PBS buffer containing 1% Tween 20 ensures that the transfer of SERRS nanotags on the NC membrane is unimpeded (Fig. S7a†). As shown in Fig. S7b and c,† the PBST buffer (1% Tween) containing 20% FBS can achieve the highest signal-to-noise ratio (SNR) on the test zones. Therefore, PBS solution (pH 7.4, 10 mM) containing 1% Tween 20 and 20% FBS was chosen as the running buffer for further experiments. Considering the effect of flow speed and signal strength of the AuNRs nanotags running on the NC membrane, we next compared three commonly used NC membranes (CN95, CN140 and HF135) with different pore sizes for AFP detection. By comparing Raman signals from the test lines of the positive and negative strips, we found the HF135 membrane exhibited the best SNR, which with a nominal pore size of 8 μm (Fig. S8a†). Thus, HF135 membrane was chosen to build the SERRS-LFIA detection system. Then, we optimized the concentration of coated antibodies on the test line, and the results showed that the SNR reach the highest when the concentration of the AFP capture antibody was 0.8 mg mL^−1^ (Fig. S8b†). Next, the amount of AuNR nanotags on the conjugate pad was optimized. Stock AuNR nanotags solution was diluted by 1-fold, 2-fold, 3-fold, and 4-fold, and then used to prepare the conjugate pad. As shown in Fig. S9,† the 2-fold dilution was found to provide the best SNR. The signal reproducibility of the assay was also investigated. Big laser spot (∼105 μm) was used to scan the test line, which is an effective way to detect more SERS nanotags-AFP immunocomplexes in one test. As illustrated in Fig. S10a,† laser scans were taken along the vertical axis and horizontal axis of test line, respectively. The SERS spectra of longitudinal line clearly show that the Raman signal decreases along the flow direction from the test line to the control line (Fig. S10b†). This phenomenon can be attributed to the front of the test line is the first contact site encountered by the nanotags, therefore formed the most sandwich immunocomplexes. Fig. S10c† shows the good signal reproducibility of five spots of the front edge region of test line (relative standard deviation [RSD] = 4.7%). Thus, in this study, five spots in the front area of the test line were measured for each sample to ensure the accuracy and signal reproducibility of the assay.

### 3.4 Quantitative analysis of AFP based on SERRS-LFIA assay

**Fig. 3:**
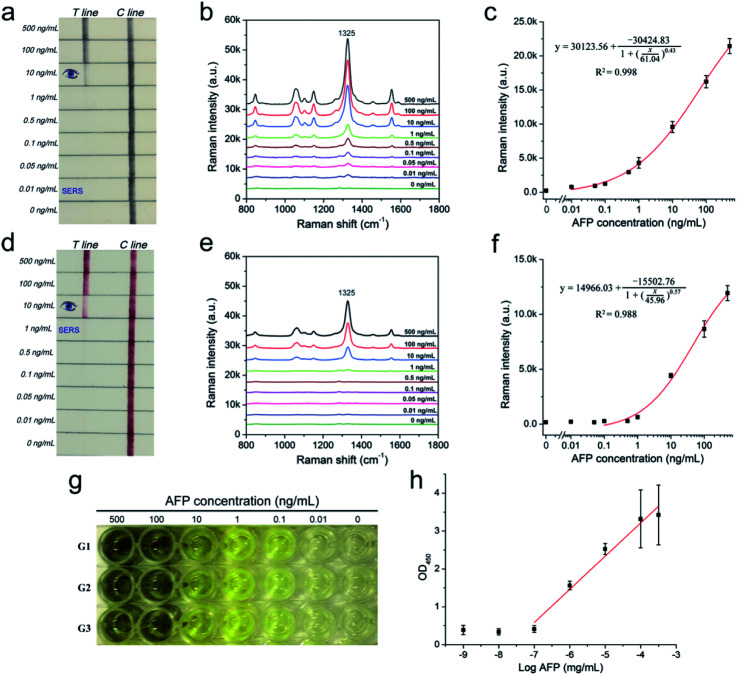
Analytical performance of the SERRS-LFIA strips. Photographs (a), SERS spectra measured in the test lines (b), and corresponding calibration curve (c) of the AuNR-based SERRS-LFIA strip for AFP detection. Photographs (d), SERS spectra measured in the test lines (e), and corresponding calibration curve (f) of the AuNP-based SERS-LFIA strip for AFP detection. The spectra were acquired at 785 nm excitation wavelength (10 mW, 10 s). (g) Colorimetric analysis for different concentrations of AFP in the ELISA plates. (h) Calibration curve obtained from the ELISA analysis. Error bars indicate the SD calculated from the three measurements.

A series of AFP samples of different concentrations was prepared and tested to evaluate the sensitivity and dynamic range of the SERRS-LFIA strip for the detection of tumor biomarkers. SERRS detection was performed in accordance with the optimal assay conditions; the detection results can be obtained in 15 min. Fig. 3a shows the photographs of the AuNR-based SERRS-LFIA strips tested at different concentrations of AFP (500 ng mL^−1^ to 0 ng mL^−1^). The dark band of AuNR nanotags on the test lines was observed at high concentration of AFP and gradually weakened with decreasing AFP concentration. Meanwhile, a dark band of the control line consistently appeared, indicating that the strip worked normally. With naked-eye observation, the visual sensitivity of the dark test line for the SERRS-strips of AFP was 10 ng mL^−1^. For the Raman mode, AFP was quantitatively analyzed through the characteristic SERRS signal of the AuNR nanotags on the test lines. Fig. 3b shows the SERRS spectra that correspond to different concentrations of AFP. Notably, the SERRS signal that originated from DTNB decreased gradually with decreasing AFP concentration. The main peak of DTNB at 1328 cm^−1^ was still visible and could be distinguished from that of the blank control, although the concentration of AFP was reduced to 0.01 ng mL^−1^. The calibration curve of the AuNR-based SERRS strip was constructed by the sigmoidal function of AFP concentrations and the SERRS intensity at 1328 cm^−1^ from their test lines (Fig. 3c). Error bars represent the SD from five independent measurements. The limits of detection (LOD) of AFP *via* the SERRS-LFIA strip was determined by the IUPAC method (LOD = *y*_blank_ + 3 × SD_blank_, where *y*_blank_ and SD_blank_ are the average SERRS intensity and standard deviation of the blank control, respectively).^28^ Based on this formula, the LOD of the assay for AFP detection was calculated to be 9.2 pg mL^−1^. To compare directly the detection performance of the SERRS-LFIA strip with that of the commonly used SERS-LFIA strip, we prepared a AuNP-based SERS-LFIA strip using the same paired AFP antibodies but replaced the AuNR nanotags with 50 nm DTNB-modified AuNPs. As shown in Fig. 3d–f, the visualization result of AuNP-based SERS strip was also observed at 10 ng mL^−1^, and the LOD of AFP with SERS signal was 1 ng mL^−1^. For comparison, the visualization sensitivity of the SERRS-LFIA strip was equal to that of the AuNP-based SERS strip, whereas the SERS sensitivity was about 100 times than that of the AuNP-based SERS strip. The marked improvement in the sensitivity of the SERRS-LFIA strip can be attributed to the LSPR of AuNR nanotags at 785 nm, which matched the excitation wavelength of the laser device and generated the strongest SERRS effect. To further compare the detectability of the SERRS-LFIA strip with traditional immunoanalytical methods, a commercial ELISA kit for AFP detection (BD Biosciences, USA) was used to detect the same AFP samples. Fig. 3g and h show the ELISA results for colorimetric analysis and the corresponding calibration line for different concentrations of the AFP sample (500 ng mL^−1^ to 0 ng mL^−1^). The detection limit of ELISA was estimated to be 0.1 ng mL^−1^ for AFP, which was more than 10 times higher than that of the SERRS-LFIA strip. Moreover, the developed SERRS-LFIA strip exhibited a wider dynamic range and shorter detection time than the ELISA analysis.

**Fig. 4:**
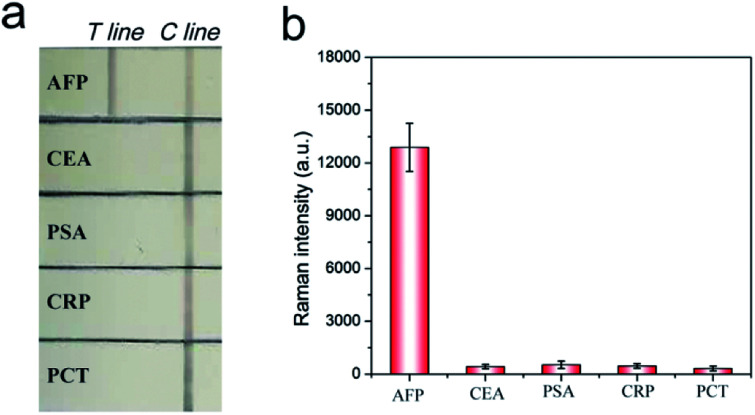
Specificity of the SERRS-LFIA strip. Photograph (a) and Raman intensity (b) of the SERRS-LFIA strip in the presence of AFP (100 ng mL^−1^) and other four biomarkers (1000 ng mL^−1^). Error bars represent the SD of three repetitive experiments.

To verify the specificity of the SERRS-LFIA strip, we tested four other protein biomarkers, including CEA, PSA, procalcitonin (PCT) and C-reactive protein (CRP), at a high concentration (1000 ng mL^−1^) as interferers. Fig. 4a shows that both the test and control lines were observed for the AFP group, whereas only the control line appeared for the other four interferers. Fig. 4b shows that these interferers exhibited no remarkable SERS intensity on the test line, whereas 100 ng mL^−1^ AFP produced a strong Raman signal. These experimental results reveal that the proposed SERRS-LFIA strip possesses good specificity and selectivity for AFP detection.

### 3.5 Detection of the AFP in biological sample

**Fig. 5:**
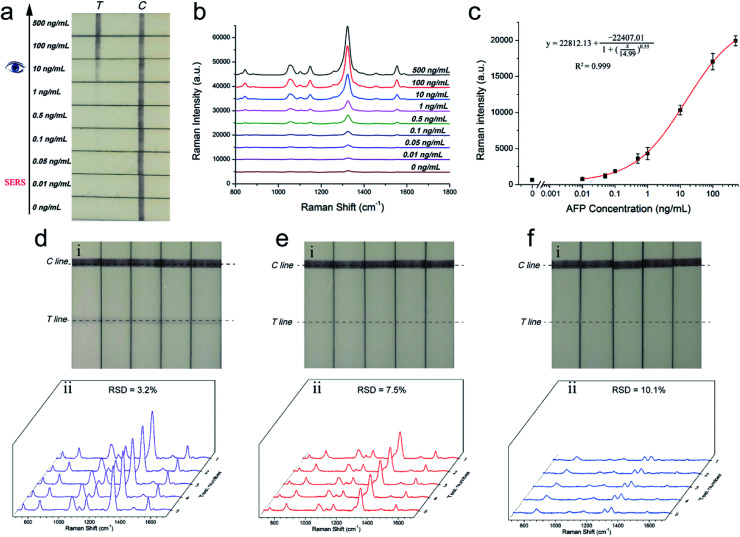
Performance evaluation of the SERRS-LFIA strip in fetal calf serum. (a) Photographs and (b) SERS spectra of AuNR nanotags-based SERRS-LFA strip for detection of AFP in fetal calf serum at concentrations ranging from 500 ng mL^−1^ to 0.01 ng mL^−1^. (c) Corresponding calibration curve of SERRS-LFIA for the concentrations of AFP. Assay reproducibility of five tests of AFP at concentrations of (d) 10, (e) 1, and (f) 0.1 ng in unprocessed fetal calf serum.

Considering the excellent stability and SERRS performance of the BSA-coated AuNR nanotags used in this assay, the SERRS-LFIA strip shows potential for direct detection of complex samples. It should be noted that AFP antigens (or other cancer biomarkers) are secreted by human cells, and exhibit a baseline concentration in human blood, which will be obviously elevated in case of HCC or other liver cancer-related disease.^47–49^ Owing to the low amount of AFP (<20 ng mL^−1^) that naturally exists in normal human blood or serum, human biological samples were excluded from this study. In the proposed SERRS-strip, a pair of mouse anti-human AFP antibodies was used as the specific antibody for human AFP detection, and wouldn't immune bind to any components in bovine serum. Thus, the assay was conducted to detect human AFP spikes in unprocessed fetal calf serum to evaluate the quantitative capability and practical application of the SERRS-LFIA strip in biological samples. Fig. 5a shows that the visual sensitivity of the SERRS-LFIA strip for AFP in fetal calf serum was 10 ng mL^−1^. As shown in Fig. 5b, their corresponding SERS intensities as read out by the portable Raman spectrometer increased markedly with the increase in AFP concentrations. The SERS signals were analyzed by plotting the peak intensity at 1328 cm^−1^ as a sigmoidal function of the AFP concentration to construct a calibration line (Fig. 5c). The LOD of AFP is 11.7 pg mL^−1^ in the fetal calf serum, and it was calculated from the sigmoidal fitting curve where the AFP concentration generated the SERRS intensity of 1328 cm^−1^. This intensity exceeded the SD of the blank control by three times. The reproducibility of the SERRS-LFIA strip in serum was also investigated by testing fetal calf serum samples containing different concentrations of AFP. Five independent tests were conducted to measure AFP samples at concentrations of 10, 1, and 0.1 ng mL^−1^. As shown in Fig. 5d–f, the RSD values of the SERRS intensity at 1328 cm^−1^ were 3.2%, 7.5%, and 10.1%, respectively, indicating high repeatability and reliability of the method in serum samples. On the basis of these results, we can conclude that the proposed method shows excellent stability in serum sample matrix, and its detection performance is unaffected. Furthermore, the SERRS-LFIA strip showed no obvious signal intensity changes in the test line after storage for 60 days under sealed condition (Fig. S11†). This result indicated the SERRS-LFIA strip has excellent long-terms stability.

To evaluate further the clinical feasibility of the SERRS-LFIA strip, we performed detection of AFP in real clinical samples. Ten clinical serum samples were received from the Affiliated Hospital of Xuzhou Medical University, and their AFP concentrations were determined by the chemiluminescent microparticle immunoassay system (CMIA). The SERRS-LFIA detection was performed according to the developed protocol, and the results were compared with those obtained by the CMIA method. As summarized in Table 1, the detection value of the clinical samples from the two methods were highly consistent. This result indicates that the SERRS-LFIA method also possesses the capability for the rapid and sensitive detection of AFP in clinical serum samples. Considering its easy-to-use format and low cost, the proposed SERRS-LFIA system showed strong potential as a POCT tool for the rapid and high-sensitivity detection of AFP in clinical samples. Notably, the SERRS-LFIA strip is an effective universal detection method, not only for AFP detection. By changing the specific detection antibodies, the SERRS-LFIA system can be directly applied to detect other biomarkers, such as tumor biomarkers (CEA, PSA, and CA15-3), inflammatory markers (PCT and CRP), and cardiac markers, in clinical serum samples.

**Table 1:** Quantification in clinical serum specimens of AFP determined by the CMIA system and the SERRS-strip system

Sample number	CMIA system (ng mL^−1^)	SERRS-LFIA system (ng mL^−1^)
1	18.66	15.2 ± 2.3
2	40.27	43.3 ± 3.6
3	747.8	728.5 ± 22.5
4	123.1	110.2 ± 11.3
5	131.8	122.1 ± 9.7
6	210.8	202.5 ± 13.1
7	46.94	40.7 ± 4.2
8	4971	>500
9	10 770	>500
10	185.2	164.3 ± 9.4

## 4. Conclusions

In this study, we have developed a rapid, sensitive, and stable SERRS-LFIA strip for AFP detection using BSA-coated AuNRs as SERRS nanotags. The designed SERRS nanotags consisted of four parts: a AuNR with plasmon resonance peak located at 785 nm as the SERRS substrate, a layer of Raman dye DTNB to generate strong SERS signals, a layer of BSA to act as a protective shell, and surface-modified AFP detection antibodies to specifically bind AFP. The SERRS nanotags were used for the first time in the LFIA system to overcome the low sensitivity of the colorimetric assay and ensure the stability in complex samples. For SERRS detection, quantitative determination of AFP can be easily realized by recording the SERRS signal of the AuNR nanotags trapped on the test line. The LOD for AFP with SERRS-LFIA strip reached 9.2 pg mL^−1^, which was 100 times more sensitive than that using the AuNP nanotag-based strip. Compared with the commercial ELISA, the proposed method is 10 times more sensitive and exhibits a wider detection range in serum samples. The specificity, stability, and potential clinical applications of the proposed method were also demonstrated. These results indicate that the SERRS-LFIA strip features potential for rapid and accurate detection of cancer biomarkers in field tests.

## Conflicts of interest

There are no conflicts to declare.

## Supplementary Material

RA-010-C9RA09471G-s001
