# Supplementary material for: Rapid, quantitative and ultra-sensitive detection of cancer biomarker by a SERRS-based lateral flow immunoassay using bovine serum albumin coated Au nanorods
Source: RSC Adv. 2020 Jan 2;10(1):271–81. doi: 10.1039/c9ra09471g (PMC9047559; doi:10.1039/c9ra09471g)
Supplement: RA-010-C9RA09471G-s001 [file RA-010-C9RA09471G-s001.pdf]

Electronic Supplementary Material (ESI) for RSC Advances.
This journal is © The Royal Society of Chemistry 2019

Electronic Supplementary Material

Rapid, quantitative and ultra-sensitive detection of cancer biomarker by a SERRS-based lateral flow immunoassay using bovine serum albumin coated Au nanorods

Luchun Lu[a,b,1], Jiangliu Yu[a,1], Xiaoxian Liu[a], Xingsheng Yang[a], Zihui Zhou[c], Qing Jin[a,*], Rui Xiao[a,b,*], Chongwen Wang[a,b,c,*]

[a] *College of Life Sciences, Anhui Agricultural University, Hefei 230036, PR China.*

[b] *Beijing Institute of Radiation Medicine, Beijing 100850, PR China.*

[c] *Anhui Provincial Key Laboratory of Veterinary Pathobiology and Disease Control, Anhui Agricultural University, Hefei 230036, PR China.*

S1 EF calculation:

To quantify the enhancement ability of the AuNRs, the enhancement factor (EF) was calculated as the ratio of photons scattered by the SERS substrate and the normal substrate. EF was estimated according to the following equation:

$EF=(I_{SERS}/I_{bulk})(N_{bulk}/N_{SERS})$, whereas N_{bulk} and N_{SERS} is the number of molecules contributed to the Raman and SERS signal, respectively, and I_{bulk} and I_{SERS} is the respective signal intensity of the related peaks. However, intrinsic EF is difficult to estimate because several variables, such as adsorbed molecules and laser scattering volume, are difficult to obtain. In our experiment, all the other parameters, including the laser diameter, laser power, exposure time, and microscopic magnification, were identical. The chemical droplets were of the same volume, and the number of detected DTNB molecules was proportional to its concentration. Therefore, the EF was roughly estimated by comparing the intensity of the Raman peak in the SERS spectrum with that in the normal Raman spectrum according to the equation:

$EF = (I_{SERS}/I_{RS}) \times (C_{RS}/C_{SERS})$, where I_{SERS} and I_{RS} are the vibration intensities in the SERS and normal Raman spectra of DTNB molecules, and C_{RS} and C_{SERS} are the concentrations of the DTNB molecules in the reference and SERS samples, respectively.

The peak at 1328 cm^{-1} from the DTNB Raman spectrum (Fig. S1) was chosen for analysis, and the intensities for peaks from Si substrate (black line) and AuNRs (red line) were 4214 and 12218 a.u., respectively. The DTNB concentrations for peaks (black line and red line) were 0.5 M and 10^{-6} M, respectively. Therefore, the EF of the AuNRs was roughly estimated to be 1.45×10^{6}. Moreover, the value of SERS intensity from 50 nm AuNPs (blue line) at the same DTNB concentration (10^{-6} M) was 2867 a.u., thus the EF of 50 nm AuNPs was calculated to be 3.4×10^{5}. These calculations supported the fact that the AuNRs based SERRS nanotags provided a higher SERS activity than commonly used AuNPs.

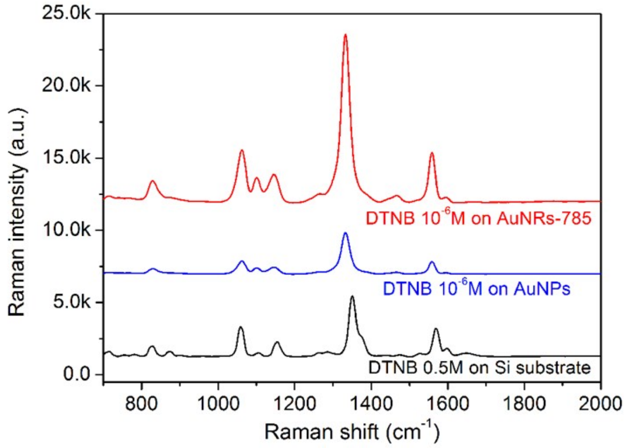

Fig. S1 Raman spectra of DTNB molecules on different substrates: 0.5 M DTNB on Si substrate (black line), 10^{-6} M DTNB on 50 nm AuNPs (blue line), and 10^{-6} M DTNB on AuNRs SERRS nanotags (red line).

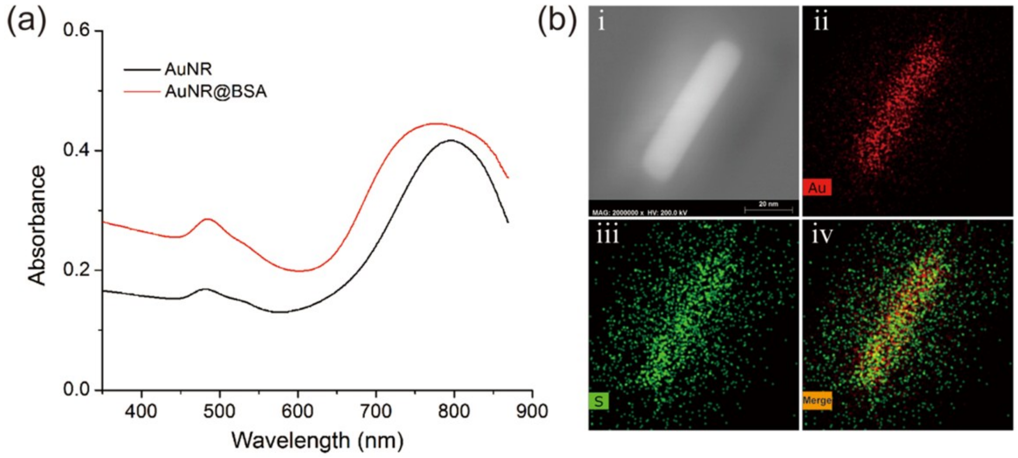

Fig. S2 (a) UV–vis spectra of AuNR (black line) and AuNR@BSA (red line). (b) EDS elemental mapping images of single AuNR@BSA.

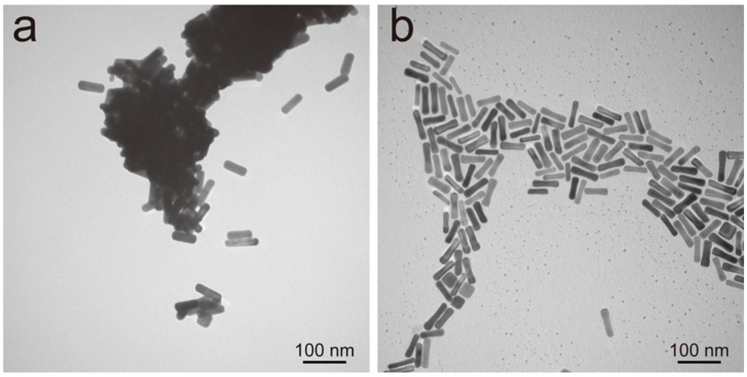

Fig. S3 TEM images of AuNR-DTNB and AuNR@BSA in the NaCl solution (1000 mM).

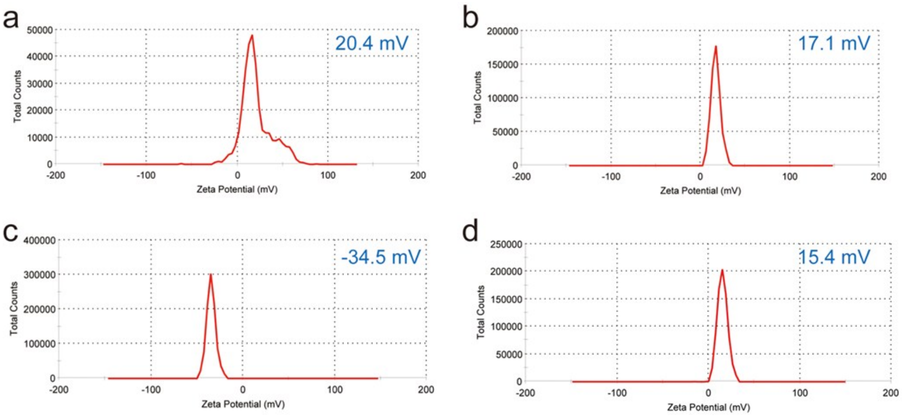

Fig. S4 Zeta potentials data of the AuNR, AuNR-DTNB, AuNR@BSA and AuNR nanotags in aqueous solution.

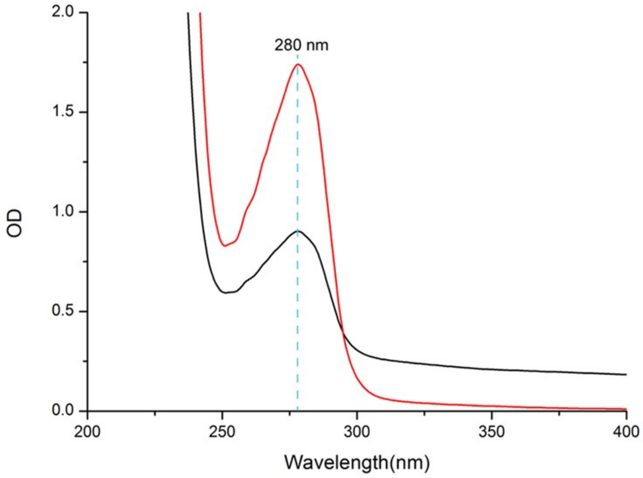

Fig. S5 Absorption spectra of antibody solution before (red line) and after (black line) binding to AuNR@BSA.

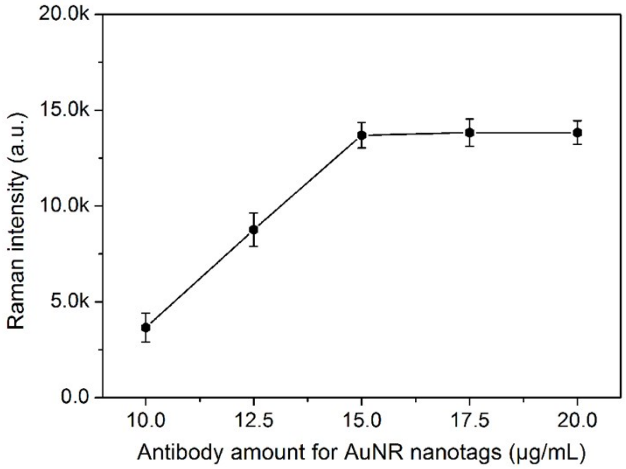

Fig. S6 SERS intensity of the test line versus the amount of AFP antibody modified on the AuNR nanotags. The error bars represent the standard deviations from 3 measurements.

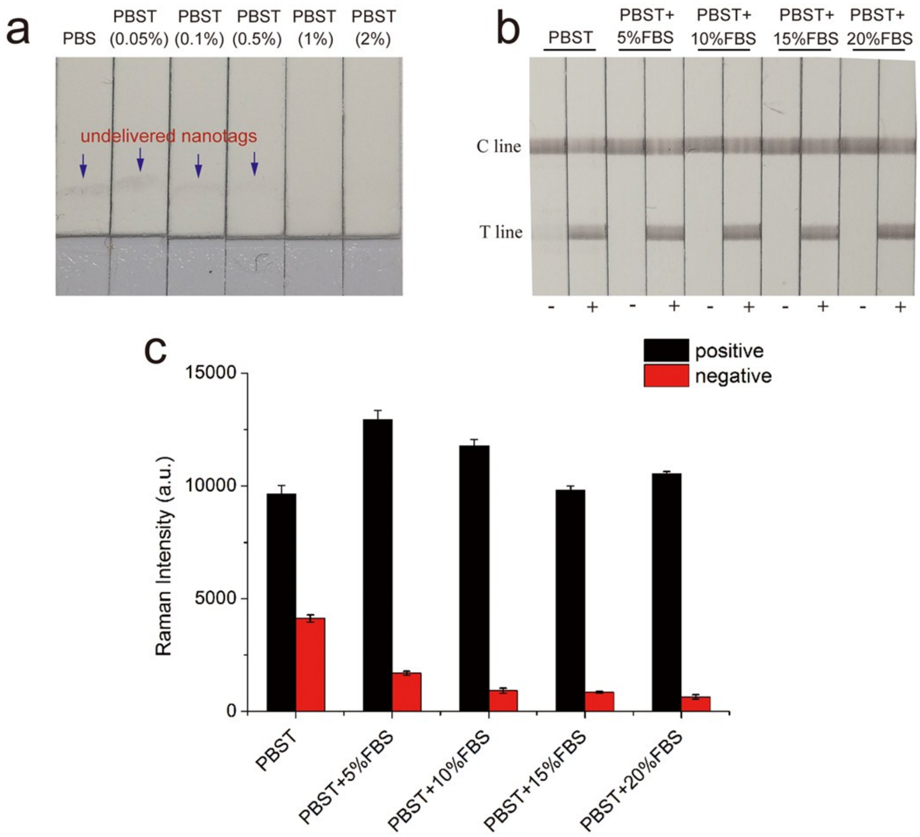

Fig. S7 Optimization of running buffer on the AuNRs-based SERRS strip. (a) Images of the test SERRS-strips at different PBST solution (containing 10 mM PBS, pH 7.4, 0-2% Tween 20). (b-c) Images and SERS intensities of SERRS-strips using different running buffers: PBST (1% Tween 20), PBST (1% Tween 20, 5% FBS), PBST (1% Tween 20, 10% FBS), PBST (1% Tween 20, 15% FBS) and PBST (1% Tween 20, 20% FBS). The error bars represent the standard deviations from five measurements.

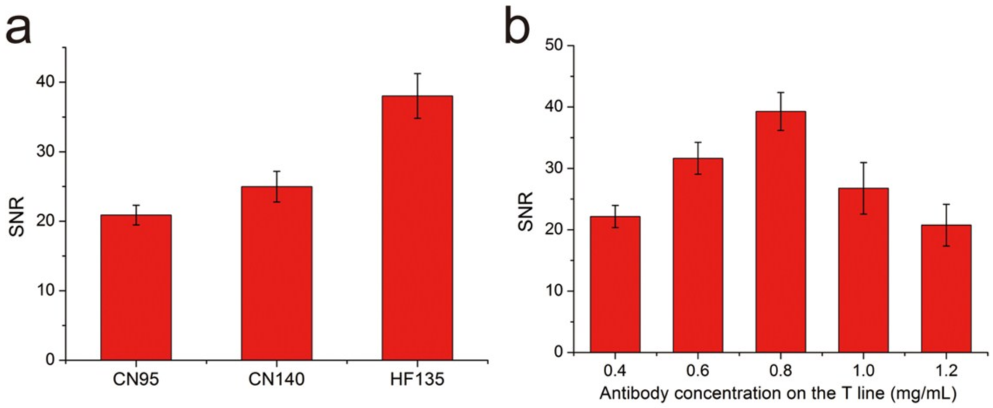

Fig. S8 Optimization of NC membrane (a) and antibody concentration on the T line (b) for the SERRS-strip. Error bars represent the standard deviation of three repetitive experiments.

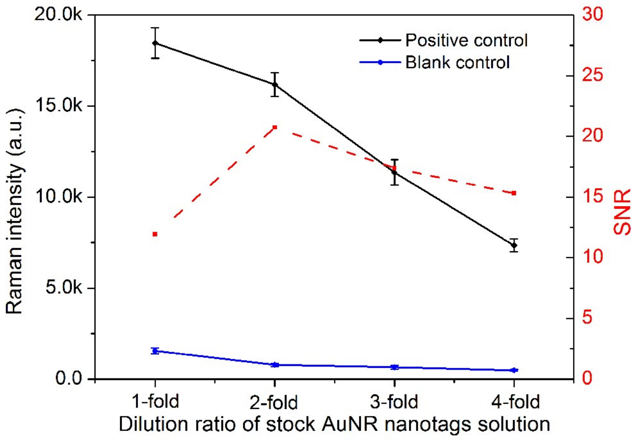

Fig. S9 Optimization of the amount of AuNR nanotags on the conjugate pad. Error bars represent the standard deviation of three repetitive experiments.

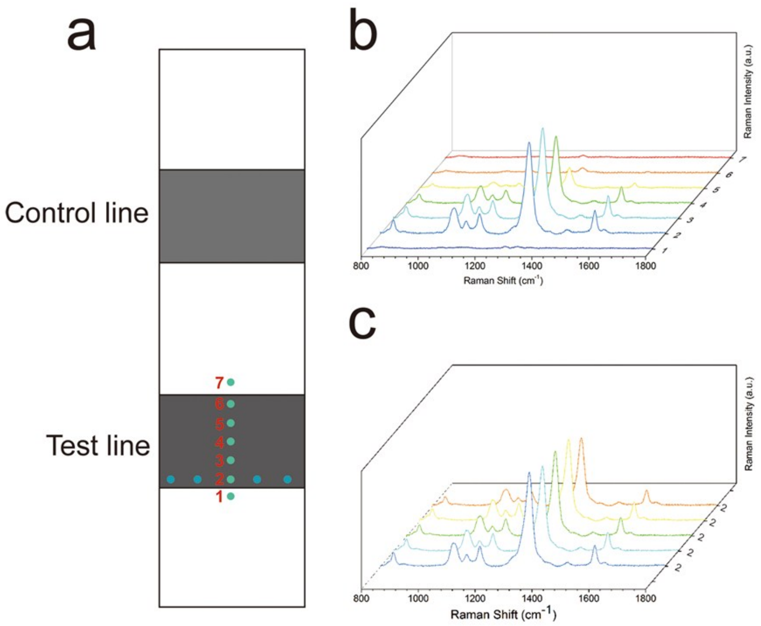

Fig. S10 The signal reproducibility of the test line of SERRS-strip. (a) Schematic illustration of a test line showing 7 different locations along the vertical axis and 5 different locations along the horizontal axis for Raman measurements. (b) SERS spectra of the 7 points on the vertical axis of the test line, and (c) SERS spectra of the 5 points on the horizontal axis of front edge region of the test line.

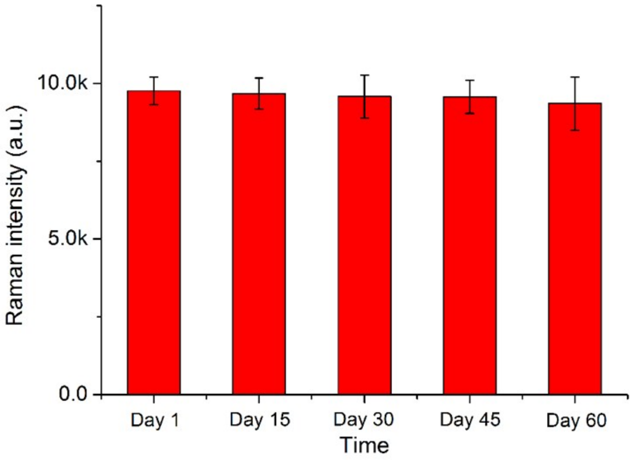

Fig. S11 Assay stability of the test line intensity for SERRS-strip stored for 60 days. Error bars are standard deviation of five repetitive experiments.
